# Milk-Sickness

**Published:** 1857-11

**Authors:** John C. Beck

**Affiliations:** Cadiz


ARTICLE V.—*Milk-Sickness.* By JOHN C. BECK, M. D., of Cadiz.

Dr. JOHN PICKARD,

DEAR SIR,—In the June No. of the "Journal" you present some items on the Pathology of Milk-Sickness that are worthy of the earnest attention of the profession. Your views so nearly coincide with my own, that I wish to add my testimony with yours. Your observations of the disease and surrounding circumstances corresponds with the experience of our practitioners in this portion of Indiana. Dr. Wilkinson has said truly, that our book men know but little of this disease, and he might have extended this remark to many other items which the profession of the Great West are compelled to rely upon from their own resources. I am satisfied that it has a vegetable origin. Came to this conclusion while investigating this subject many years ago, when such large sums were offered as rewards for the discovery of its cause. Prof. Mitchel has given the subject the clearest exposition of the vegetable theory on record, and Dr. W.'s newspaper sketch is very suggestive of its vegetable origin.

If physicians in each section of our country would arm themselves with a complete outfit of botanical knowledge, and observe closely the kinds of fungi that grow in their neighborhoods, and especially in the "small lots" where it is known to exist, we would soon be able to pick it out of the thousands of kindred herbs, and give it to animals, and be able to determine satisfactorily by experiment the precise species that causes the disease.

You perhaps recollect its activity in some parts of Kentucky a few years ago, and that it prevailed exclusively in winter. Here it prevails in all seasons except winter, and the character of the spring determines its appearance early or late. If we have abundance of rain in the spring, with hot sunshine alternating with thunder-storms and growing showers, as we sometimes have the last of April and during May and June, then we confidently expect Milk-Sickness in May and June. A dry spring and summer will postpone it till September or October. These are facts well known in this part of the country.

Of the symptoms. Those most frequently met with are the peculiar odor, vomiting, inordinate pulsation of the abdominal aorta, thirst, craving cold drinks (which our good old fathers formerly forbid under severe cautionings); and the post-mortem are found to be enteritis, which was active congestion, perhaps for the first few days; even the jejunum is inflamed in some cases, and the mucous lining of the ileum in nearly all were in a high state of inflammation. I have bled, used fomentations, and gave cold drinks, and large doses of quinine dissolved in whiskey. About two-thirds of the cases have received the above treatment, with some unimportant variations. Commenced bleeding and giving quinine about September, 1845, and have since been in the habit of relying on such remedies as are useful in congestive remittent fever.

